# An effectiveness comparison of acupuncture treatments for insomnia disorder: A bayesian network meta-analysis protocol

**DOI:** 10.1097/MD.0000000000012060

**Published:** 2018-08-21

**Authors:** Jing Chen, Liming Lu, Nenggui Xu, Jun Chen, Yupeng Fan, Fen Feng, Xiaolan Qin, Yu Kui

**Affiliations:** a Guangdong Provincial Hospital of Chinese Medicine, Guangdong Provincial Academy of Chinese Medical Sciences, The Second Clinical College of Guangzhou University of Chinese Medicine; b Guangzhou University of Chinese Medicine, Guangzhou; c Chengdu University of Traditional Chinese Medicine, Chengdu, China.

**Keywords:** acupuncture, Chinese medicine, insomnia, meta-analysis, network

## Abstract

**Background:**

Acupuncture (ACU) is used frequently in the management of insomnia disorder in China. Whereas there is variability in practice regarding the selection of ACU treatments, most choices are made based on personal experience or preference of clinician. This study uses network meta-analysis to compare the effectiveness of different forms of ACU for insomnia and assesses the evidence with the Grading of Recommendations Assessment, Development, and Evaluation (GRADE) approach.

**Methods:**

A comprehensive search for randomized controlled trials (RCTs) of ACU treatments for insomnia disorder will be carried out in PubMed, Embase, Cochrane Library, China BioMedical Literature (CBM), China National Knowledge Infrastructure (CNKI), Chongqing VIP (CQVIP), and Wanfang, from their inceptions to April 2018. The quality of the included RCTs will be evaluated with the risk of bias (ROB) tool and evidence will be evaluated by GRADE. STATA 13.0 and WinBUGS 1.4.3 through the GeMTC package will be used to perform a network meta-analysis to synthesize direct and indirect evidence.

**Results:**

The results of this network meta-analysis (NMA) will be submitted to a peer-reviewed journal for publication.

**Conclusion:**

The results of our study will provide the best possible ACU choice for clinicians, and the best possible strength of the evidence with the GRADE approach.

## 1 Introduction

According to the Diagnostic and Statistical Manual of Mental Disorders, Fifth Edition (DSM-5), approximately one-third of adults report insomnia symptoms, 10% to 15% experience impairment in social and daily life, and 6% to 10% have symptoms that could be diagnosed as insomnia disorder.^[1]^ Long-term insomnia often contributes to cognitive impairment, which may inhibit the ability to perform routine tasks. A cross-sectional telephone survey^[2]^ of 7428 workers in the US estimated annual insomnia-related work losses of 367 million days and $91.7 billion. Meanwhile, insomnia may increase the risk of certain mental illnesses and other diseases, leading to increased medical expenditures. Roughly two-third of the above losses are caused by comorbidity (26 comorbid diseases) and its related annual costs were $63.2 billion and 252.7 million days.^[2]^

Generally, treatments for insomnia are divided into nonpharmacologic and pharmacologic therapies. Cognitive behavioral therapy (CBT) is recommended as a first-line treatment by the Australasian Sleep Association (ASA). However, access to treatment is not always affordable or available^[3,4]^ because there are few qualified CBT therapists in China^[5]^ and consultations are usually expensive.^[6]^ Although there is insufficient evidence on the safety of the pharmacological treatments,^[7]^ observational studies have shown that hypnotic drugs may be associated with infrequent, but serious, adverse effects such as dementia, serious injury, and fractures.^[8–11]^

Several recent studies of acupuncture (ACU) for insomnia have shown optimistic results. Liu^[12]^ systematically evaluated 15 studies on the efficacy of ACU for insomnia. Interventions included manual ACU, electro-ACU, moxibustion, auriculotherapy, Chinese medicine, and Western medicine; Comparators included Chinese medicine and Western medicine. Most of the included studies report the Pittsburgh Sleep Quality Index (PSQI), but few describe the individual components of the PSQI. Meta-analysis has shown that ACU or ACU combined with Chinese/Western medicine has a better effect on the total PSQI score than Chinese medicine or Western medicine alone. Among the studies, only 1 reported adverse events without detailed information. However, the authors stated that because most of the included studies were of low methodology quality (including the application of randomization, allocation concealment and blinding) the above results should be taken with caution.

Although ACU is frequently used for the management of insomnia disorder in China, there is variability in practice regarding the choice of ACU treatments. Most choices are made based on the personal experience or preference of clinicians. Therefore, we plan to use network meta-analysis (NMA) to compare the effectiveness of the following 10 ACU treatments recommended by authorized Chinese medicine textbooks,^[13–15]^ clinical guidelines^[16–18]^ and acupuncturists: manual ACU, electro-ACU, moxibustion, auriculotherapy, bloodletting therapy, cupping, catgut embedding, intradermal needling, acupoint application, and plum-blossom needle. The quality of evidence for outcomes will be simultaneously assessed with the Grading of Recommendations Assessment, Development, and Evaluation (GRADE) approach. We hope that the results of our study will provide not only the best possible choice of ACU for clinicians, but also the strength of the evidence using GRADE.

## 2 Methods

This is a systematic review and ethical approval was not necessary.

### 2.1 Study registration

The study protocol has been registered on PROSPERO CRD42017067402.(https://www.crd.york.ac.uk/PROSPERO/).

### 2.2 Eligibility criteria

#### 2.2.1 Type of study

All randomized controlled trials that investigate the effectiveness of ACU treatments for insomnia disorder will be included.

#### 2.2.2 Participants

Patients must be over 18, any gender, disease stage or severity, and either with or without traditional Chinese medicine (TCM) syndrome differentiation. Insomnia disorder must have been diagnosed in line with at least one of the international or domestic authorized diagnostic criteria.

#### 2.2.3 Interventions

ACU treatments (as mentioned above) and pharmacotherapies; 1 ACU treatment, multiple ACU treatments, or ACU treatments (1 or more) combined with pharmacotherapies. Pharmacotherapies include drugs recommended in international or domestic authorized clinical guidelines. Studies which combine ACU treatments with pharmacotherapy are required to use the same pharmacotherapy in both the intervention and the comparator groups.

#### 2.2.4 Outcomes

The primary outcome is PSQI.^[19,20]^ In scoring the PSQI, 7 components are scored from 0 (no difficulty) to 3 (severe difficulty). The component scores are summed to produce a global score ranging from 0 to 21. Higher scores indicate worse sleep quality.

The second outcome is Insomnia Severity Index (ISI). ISI is a self-reported questionnaire that assesses the nature, severity, and impact of insomnia.^[21,22]^ Seven items are included, and each is scored from 0 (no problem) to 4 (very severe problem), producing a total score ranging from 0 to 28. Higher scores indicate severe sleep problems.

#### 2.2.5 Data source

A comprehensive search for randomized controlled trials of ACU treatments for insomnia disorder will be conducted covering PubMed, Embase, Cochrane Library, China BioMedical Literature (CBM), China National Knowledge Infrastructure (CNKI), Chongqing VIP (CQVIP), and Wanfang from their inceptions to April 2018. Language will be restricted to English and Chinese. References from the included studies and systematic reviews will also be searched. Additionally, clinical trial registries will be searched to determine the clinical trials being performed or completed.

Search strategies will be performed as outlined in the Cochrane Handbook for Systematic Reviews of Interventions Version 5.1.0.^[23]^ The search terms will be divided into 3 parts: disease, intervention, and study design. Subject headings [PubMed (MeSH), Embase (Emtree)], synonyms, and near-synonyms will be used in the retrieve, and all search strategies will be identified after multiple pre-search. The search strategy for each database will vary based on its unique characteristics.

#### 2.2.6 Study selection

Data screening and extraction will be conducted with EndNote X6, EpiData 3.1, and Excel 2013. Two independent reviewers will screen the study titles and abstracts after removing duplicates both electronically and manually in EndNote. Relevant studies consistent with the eligibility criteria will then be coded and retrieved for full-text screening with EpiData and Excel. Any disagreement between reviewers will be resolved through discussion with a third reviewer. The complete process will be presented in a PRISMA flow chart (available for download at http://www.prisma-statement.org).

Information extracted from included studies will include: literature characteristics (author, journal, year of publication, country where the study was performed, funding sources, etc.); participant information (age, gender, diagnose criteria, disease duration or stage, sample, number of withdrawals, exclusions, etc.); intervention information (details of intervention and control, treatment duration, frequency of use, follow-up period, and adverse events, etc.); and outcome (definition used in study, unit of analysis, time points collected, subgroup analysis, etc.).

#### 2.2.7 Risk of bias

The quality of the studies will be assessed with the Cochrane Collaboration Risk of Bias (ROB) Tool.^[24]^ Six domains will be included: random sequence generation, allocation concealment, blinding of participants and personnel, blinding of outcome assessment, incomplete outcome data, and selective outcome reporting. Each entry will be assessed with a judgment of “low risk”, “high risk”, or “unclear risk”. In addition, a detailed description will be provided in support of the judgment. Two independent reviewers will perform the ROB for the included studies. Any discrepancies will be resolved through discussion with a third reviewer.

#### 2.2.8 Statistical analysis

##### 2.2.8.1 Pairwise meta-analysis

Numerical variables will be presented as standardized mean difference (SMD) with a 95% confidence interval (CI). The heterogeneity of each pairwise comparison will be tested by an I^2^ statistic (test level α = 0.1). If there is no heterogeneity, a fixed-effects model will be used. If an I^2^ value is >50%, meta-regression or subgroup analysis will be used to explore possible sources of heterogeneity, provided there is a sufficient number of included studies. When there is no explanation for statistical heterogeneity, a random-effects model will be used with a test level of α = 0.05. Sensitivity analysis will also be performed to enhance the credibility of the results. Publication bias will be evaluated with a funnel plot and qualitative judgment of graph symmetry. Quantitative methods such as Begg test^[25]^ and Egger test^[26]^ will be used to help evaluate publication bias when applicable.

##### 2.2.8.2 Network meta-analysis

STATA 13.0 and WinBUGS 1.4.3 through the GeMTC package will be used to perform NMA to synthesize direct and indirect evidence. The NMA will be undertaken primarily in WinBUGS using the Markov chain Monte Carlo (MCMC) method.^[27]^ Convergence of the simulations will be evaluated with potential scale reduction factor (PSRF) and Gelman–Rubin–Brooks plots.^[28]^ The selection of the final model will depend on the deviance information criterion (DIC) value. Generally, a model with a smaller DIC value is better.^[29]^ Numerical variables will be presented as standardized mean differences (SMD) with 95% credible intervals (Cr I). The rank of treatments for each outcome will be conducted as surface under the cumulative ranking curve (SUCRA). The evidence relationship of included studies will be figured out by STATA. If there is a “closed loop,” the node splitting method will be used to evaluate the inconsistency of each loop.^[30,31]^

##### 2.2.8.3 Quality of evidence

The quality of evidence for the main outcomes will also be assessed with the GRADE approach.^[32]^ This assessment will be carried out through the Guideline Development Tool (GRADEpro GDT, https://gradepro.org/).

## 3 Discussion

Although numerous studies have assessed the effectiveness of ACU treatments on insomnia disorder, the remains a lack of evaluations and comparisons of the various treatments. As far as we know, no NMAs comparing the effectiveness of ACU treatments for the management of insomnia disorder have been published in recent years. The results of an NMA may provide a possible ranking for ACU treatments for insomnia disorder. In addition, the quality of evidence for the main outcomes will be assessed with the GRADE approach. We hope that the results will provide the best possible choice of ACU treatments for clinicians, and initiative research directions. This protocol has been developed in accordance with the preferred reporting items for systematic reviews and meta-analysis protocols and has been registered with international prospective register of systematic reviews (PROSPERO). The NMA report will be based on the PRISMA extension statement for reporting of systematic reviews incorporating network meta-analyses of healthcare interventions. Although a comprehensive search will be performed in this study, languages other than Chinese and English will not be searched, and this will lead to some bias.

## Author contributions

**Data curation:** Jun Chen.

**Formal analysis:** Liming Lu.

**Funding acquisition:** Yu Kui.

**Project administration:** Nenggui Xu.

**Supervision:** Yupeng Fan.

**Validation:** Fen Feng, Xiaolan Qin.

**Writing – original draft:** Jing Chen, Yu Kui.
